# Uncovering Interfacial Oxygen‐Bridged Binuclear Metal Centers of Heterogenized Molecular Catalyst for Water Electrolysis

**DOI:** 10.1002/advs.202417607

**Published:** 2025-03-30

**Authors:** Zhou Yu, Jian‐Ping Li, Xian‐Kun Xu, Zhong‐Chen Ding, Xiao‐Hui Peng, Yi‐Jing Gao, Qiang Wan, Ju‐Fang Zheng, Xiao‐Shun Zhou, Ya‐Hao Wang

**Affiliations:** ^1^ Key Laboratory of the Ministry of Education for Advanced Catalysis Materials Institute of Physical Chemistry College of Chemistry and Materials Science Zhejiang Normal University Jinhua 321004 P. R. China; ^2^ Zhejiang Engineering Laboratory for Green Syntheses and Applications of Fluorine‐Containing Specialty Chemicals Institute of Advanced Fluorine‐Containing Materials Zhejiang Normal University Jinhua 321004 P. R. China

**Keywords:** copper‐bipyridine complexes, heterogenized molecular catalyst, in situ Raman spectroscopy, oxygen evolution reaction, spectroelectrochemistry

## Abstract

The success of different heterogeneous strategies of organometallic catalysts has been demonstrated to achieve high selectivity and activity in photo/electrocatalysis. However, yielding their catalytic mechanisms at complex molecule‐electrode and electrochemical interfaces remains a great challenge. Herein, shell‐isolated nanoparticle‐enhanced Raman spectroscopy is employed to elucidate the dynamic process, interfacial structure, and intermediates of copper hydroxide‐2‐2′ bipyridine on Au electrode ((bpy)Cu(OH)_2_/Au) during the oxygen evolution reaction (OER). Direct Raman molecular evidences reveal that the interfacial (bpy)Cu(OH)_2_ oxidizes into Cu(III) and bridges to Au atoms via oxygenated species, forming (bpy)Cu(III)O_2_‐Au with oxygen‐bridged binuclear metal centers of Cu(III)‐O‐Au for the OER. As the potential further increases, Cu(III)‐O‐Au combines with surface hydroxyl groups (*OH) to form the important intermediate of Cu(III)‐OOH‐Au, which then turns into Cu(III)‐OO‐Au to release O_2_. Furthermore, in situ electrochemical impedance spectroscopy proves that the Cu(III)‐O‐Au has lower resistance and faster mass transport of hydroxy to enhance OER. Theoretical calculations reveal that the formation of Cu(III)‐O‐Au significantly modify the elementary reaction steps of the OER, resulting in a lower potential‐determining step of ≈0.58 V than that of bare Au. This work provides new insights into the OER mechanism of immobilized‐molecule catalysts for the development and application of renewable energy conversion devices.

## Graphical abstract

In situ Raman monitoring of an electrochemically induced interfacial oxygen‐bridged Cu(III)‐O‐Au binuclear center in heterogenized molecular catalysts, could combine surface hydroxyl groups to form the important intermediate of Cu(III)‐OOH‐Au, which then turns into Cu(III)‐OO‐Au to release O_2_. This significantly modifies the elementary reaction steps and lowers the overpotential for oxygen evolution reaction.

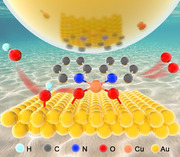

## 1 Introduction

The main obstacle to large‐scale hydrogen production through electrochemical water splitting is the cost and efficiency of current catalysts for the oxygen evolution reaction (OER).^[^ ^1^ ^]^ Extensive research on water oxidation catalysts has shown that molecular catalysts have broad application prospects because of their high activity and tunability as well as feasible integration.^[^ ^2^ ^]^ Among them, Cu complexes with sulfonated phthalocyanine or bipyridyl have been recently reported to be efficient, low‐cost, and environmentally friendly OER catalysts.^[^ ^3^ ^]^ For example, copper–bipyridine–hydroxo complexes formed at pH 11.8–13.3 have a turnover frequency of ≈100 s^−1^ for the OER.^[^ ^3b^ ^]^ A bioinspired trinuclear copper molecular catalyst developed by Zhang and co‐authors for water oxidation exhibited an impressive turnover frequency of up to 20 000 s^−1^ in a sodium bicarbonate solution.^[^ ^3c^ ^]^

However, a critical issue for molecular catalysts in the presence of first‐row transition metal complexes is their stability under the harsh conditions of the OER.^[^ ^4^ ^]^ Even if the ligand is stable, for example, copper(II) is a labile ion and can quickly undergo decoordination or oxidation.^[^ ^5^ ^]^ For example, many copper(II) complexes have been reported to form copper oxides or copper ions.^[^ ^6^ ^]^ Several papers have also shown that copper oxides, rather than their molecular precursors, are effective catalytic species. Although some molecular copper catalysts have been demonstrated to have impressive activity and durability in OER, the key active intermediates are difficult to isolate and identify, resulting in the mechanism being still not fully understood.^[^ ^7^ ^]^ On the other hand, recent extensive studies have shown that heterogeneous strategies for immobilizing molecular catalysts onto solid supports^[^ ^8^ ^]^ can their stability and activity toward the electrochemical OER. Because it can optimize the exposure of active sites, regulate the electronic structure, enhance mass and charge transfer, and stabilize the catalyst structure. Based on the understanding of their structure‐activity relationships, such catalysts can be more rationally modified and designed. However, a comprehensive understanding of the catalytic mechanisms at complex molecule‐electrode and electrochemical interfaces remains a great challenge.

**Scheme 1:**
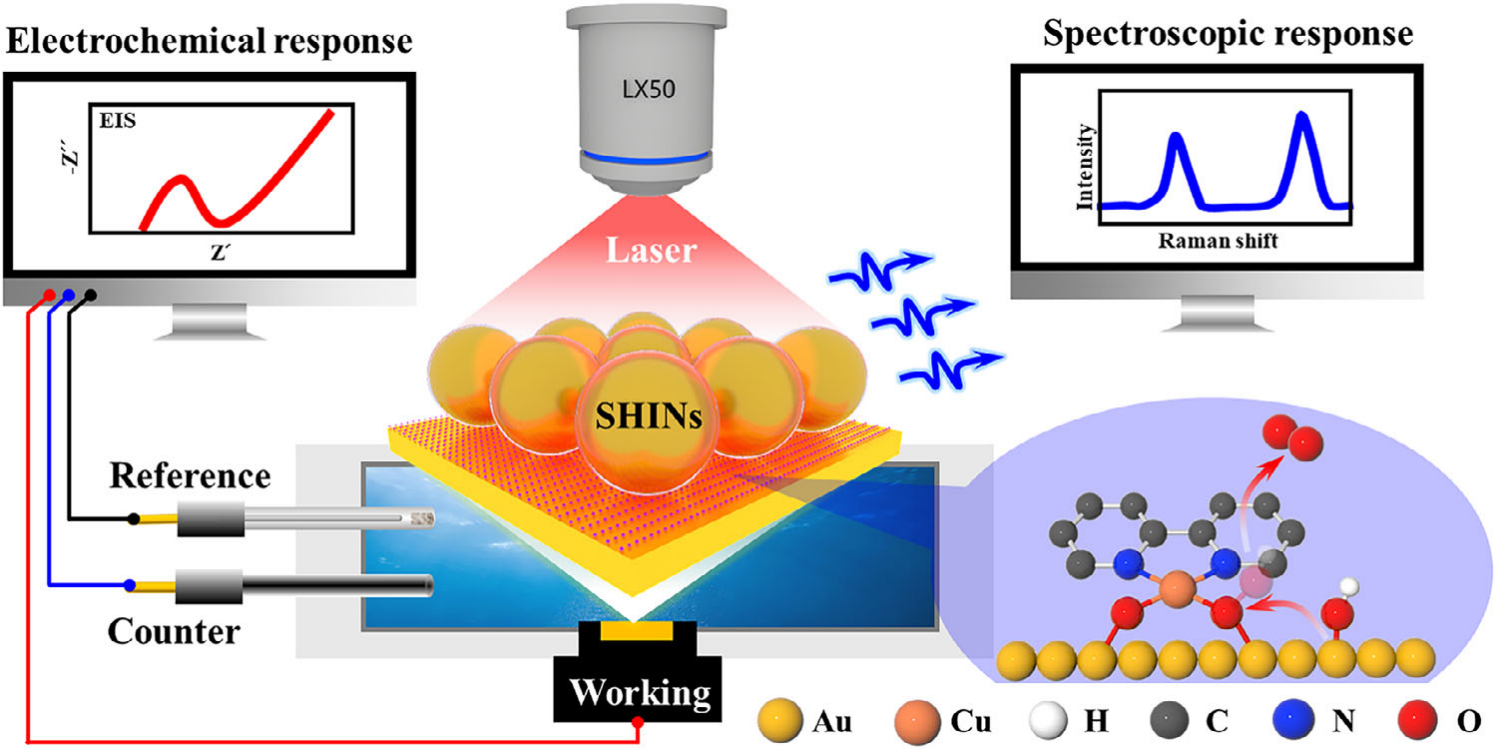
Schematic diagram of in situ monitoring of the OER process by EIS and Raman spectroscopy.

In this work, in situ shell‐isolated nanoparticle‐enhanced Raman spectroscopy (SHINERS)^[^ ^9^ ^]^ and electrochemical impedance spectroscopy (EIS) were employed to probe the dynamic process, interfacial structure and intermediates of heterogeneous molecular catalysts during electrocatalytic water oxidation (**Scheme** **1**). We prepared a copper hydroxide‐2‐2′ bipyridine ((bpy)Cu(OH)_2_) complex as a model molecular catalyst by controlling the pH of the solution. Linear‐sweep voltammetry (LSV) clearly shows that the (bpy)Cu(OH)_2_‐modified Au electrode (denoted as (bpy)Cu(OH)_2_/Au)) has a lower onset potential and larger current density for the OER than the bare Au or the use of a glassy carbon (GC) electrode (denoted as (bpy)Cu(OH)_2_/GC). Then, in situ, Raman and EIS are used to systematically probe the reaction kinetics, interfacial structure, and intermediates to clarify the enhanced OER activity and mechanism. Furthermore, in line with the direct molecular evidence in potential‐dependent EIS and SHINERS, theoretical calculations are also used to compare the reaction energy barrier of the elementary reaction steps and overpotential of the potential‐determining step during the OER. This work offers theoretical and experimental support for the advancement and industrial implementation of the OER by using heterogeneous molecular catalysts.

## 2 Results and Discussion

**Figure 1:**
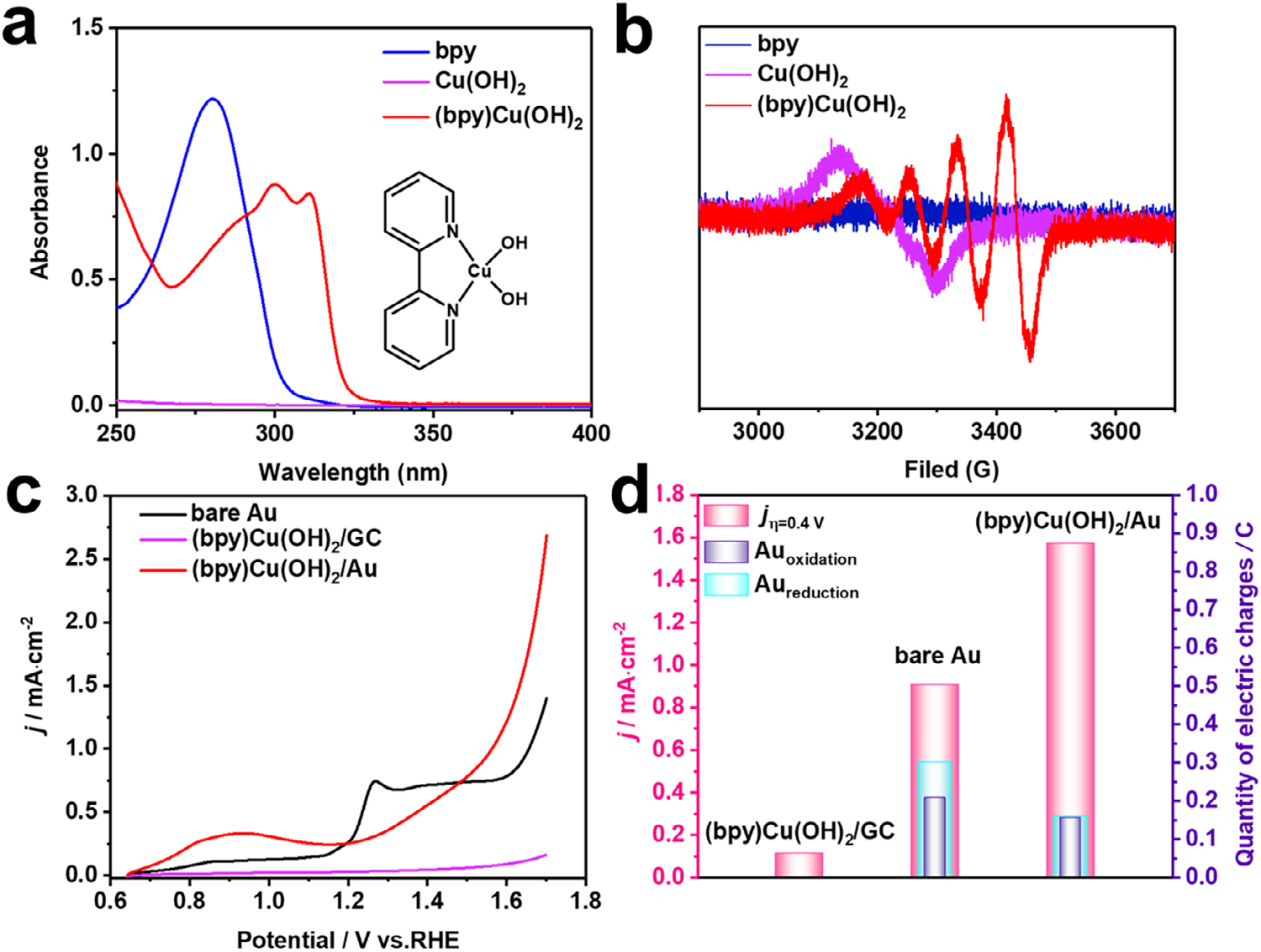
(a) UV‐vis spectra and (b) EPR spectra of a 1 mm bpy aqueous solution (blue), a 1 mM Cu(OH)_2_ aqueous solution (purple), and a 1 mm (bpy)Cu(OH)_2_ (red) aqueous solution with a pH of 12.5. (c) LSVs of the OER on the bare Au electrode (black), (bpy)Cu(OH)_2_/GC electrode (purple), and (bpy)Cu(OH)_2_/Au electrode (red) at pH = 12.5. The scan rate was 100 mV s^−1^. (d) Plots of *j* at an *η* of 0.4 V compared with the electric charges of the Au(OH)_3_ oxidation anode peak and the gold oxide reduction cathode peak for the bare Au electrode, (bpy)Cu(OH)_2_/GC (purple) and (bpy)Cu(OH)_2_/Au.

The water‐soluble molecular catalyst (bpy)Cu(OH)_2_ was prepared by controlling the solution pH to 12.5 according to previous reports.^[^ ^3^, ^10^ ^]^ Consistent with reported results,^[^ ^11^ ^]^ the intense absorbance peak at ≈280 nm is assigned to the π‐π* transition of bpy, whereas the peak at 310 nm is ascribed to the copper‐bpy charge transfer transition in the UV‐vis spectra (**Figure** **1**a). The four peaks (quartet) and the g tensors of (bpy)Cu(OH)_2_ in EPR spectra (Figure 1b; Figure S1, Supporting Information) also indicate that the copper cation was coordinated with bpy in the square‐planar or square‐pyramidal mode.^[^ ^3^, ^12^ ^]^

Next, we compared the linear sweep voltammetry curves (LSVs) of the Au or glass carbon (GC) electrodes in 100 mm NaOH + 100 mm NaOAc solution containing 1 mm (bpy)Cu(OH)_2_ (pH of ≈12.5). As shown in Figure 1c, (bpy)Cu(OH)_2_/Au exhibited significantly improved OER activity with the lowest overpotential (denoted as *η*) and largest current density (denoted as *j*) compared with those of (bpy)Cu(OH)_2_/GC and bare Au. The *j* of (bpy)Cu(OH)_2_/Au at *η* = 0.4 V is ≈14.2 times greater than that of homogeneous catalysis on a GC electrode. A 71‐fold improvement in activity could also be observed at a low scan rate of 1 mV s−^1^ in Figure S2 (Supporting Information). It is well known that the nitrogen atoms and π electrons of pyridine rings can interact with Au, which could improve the OER activity.^[^ ^9e^ ^]^

In addition, the background current primarily arises from the Faradaic processes of Cu^II^/Cu^III^ oxidation and Au oxidation in alkaline solution, there is a broad anodic peak at ≈0.9 V for (bpy)Cu(OH)_2_/Au, which can be attributed to Cu^II^/Cu^III^ oxidation according to previous reported result^[^ ^13^ ^]^ of Cu−peptoid complex with 2,2′‐bipyridine ligand and Raman spectroscopic evidence discussed in the next part. This also indicates that when the OER subsequently occurs, the copper in the catalyst is trivalent. Previous results have proven that Cu(III) is an active substance that can promote the OER.^[^ ^14^ ^]^ On the other hand, there is another broad anodic peak at ≈1.4 V for bare Au electrode. Previous reports assigned this peak to the oxidation of Au(OH)_3_ to AuOOH (Au(OH)_3_ + Au + H_2_O → 2Au*OOH* + 3(H^+^ + e^−^)).^[^ ^15^ ^]^ This AuOOH is unstable and could dehydrate into Au_2_O_3_ during water oxidation, which could reduce the contact area with water and inhibit the further catalytic cycle.^[^ ^15a^ ^]^ Interestingly, such an anodic peak becomes much less distinguishable and overlaps with the OER surge current density at (bpy)Cu(OH)_2_/Au.

Figure 1d shows the quantity of electric charges of the anodic peak from 1.24 to 1.54 V and the cathodic peak of gold oxide reduction from 0.89 to 1.19 V in Figure S3 (Supporting Information). This clearly shows that (bpy)Cu(OH)_2_/Au can effectively reduce the formation of AuOOH and all gold oxides by 21% and 41%, respectively. Therefore, the synergistic effect of the molecular catalyst on the Au electrode might promote the conversion of OH^‐^ into O_2_ and inhibit the formation of AuOOH to improve the OER performance. Controlled potential electrolysis operated at 1.64 V for 5.5 h also proves a good stability of (bpy)Cu(OH)₂/Au (Figure S4, Supporting Information).

To elucidate the catalytic process of these heterogenized molecular catalysts, the advanced Raman technique of SHINERS has been employed for in situ monitoring of interfacial structures and intermediates during the OER.^[^ ^16^ ^]^ The prepared nanoparticles consisting of an Au core with a diameter of ≈120 nm and an ≈2 nm SiO_2_ shell are pipetted onto the Au electrode as Raman signal amplifiers (Figure S5, Supporting Information). Figure S6 (Supporting Information) shows the Raman spectra of (bpy)Cu(OH)_2_/Au and bpy/Au in solution at the open‐circuit potential. Consistent with previous reports,^[^ ^17^ ^]^ the obvious Raman bands at 350 to 800 cm^−1^, 990 to 1080 cm^−1^, and 1290 to 1340 cm^−1^ are attributed to the in‐plane ring deformation, ring breathing mode, and inter‐ring stretching mode of bpy, respectively. Notably, these Raman bands of (bpy)Cu(OH)_2_/Au are significantly shifted to lower wavenumbers compared to isolated molecules in solution (Figure S6, Supporting Information). In addition, the coordination of Cu^II^ reduces the d‐π interactions between the bpy ring and Au electrode, which leads to a redshift of Raman bands for (bpy)Cu(OH)_2_ compared to bpy/Au. These confirm that (bpy)Cu(OH)_2_ immobilizes on the Au electrode via the interaction of the bpy ring.

**Figure 2:**
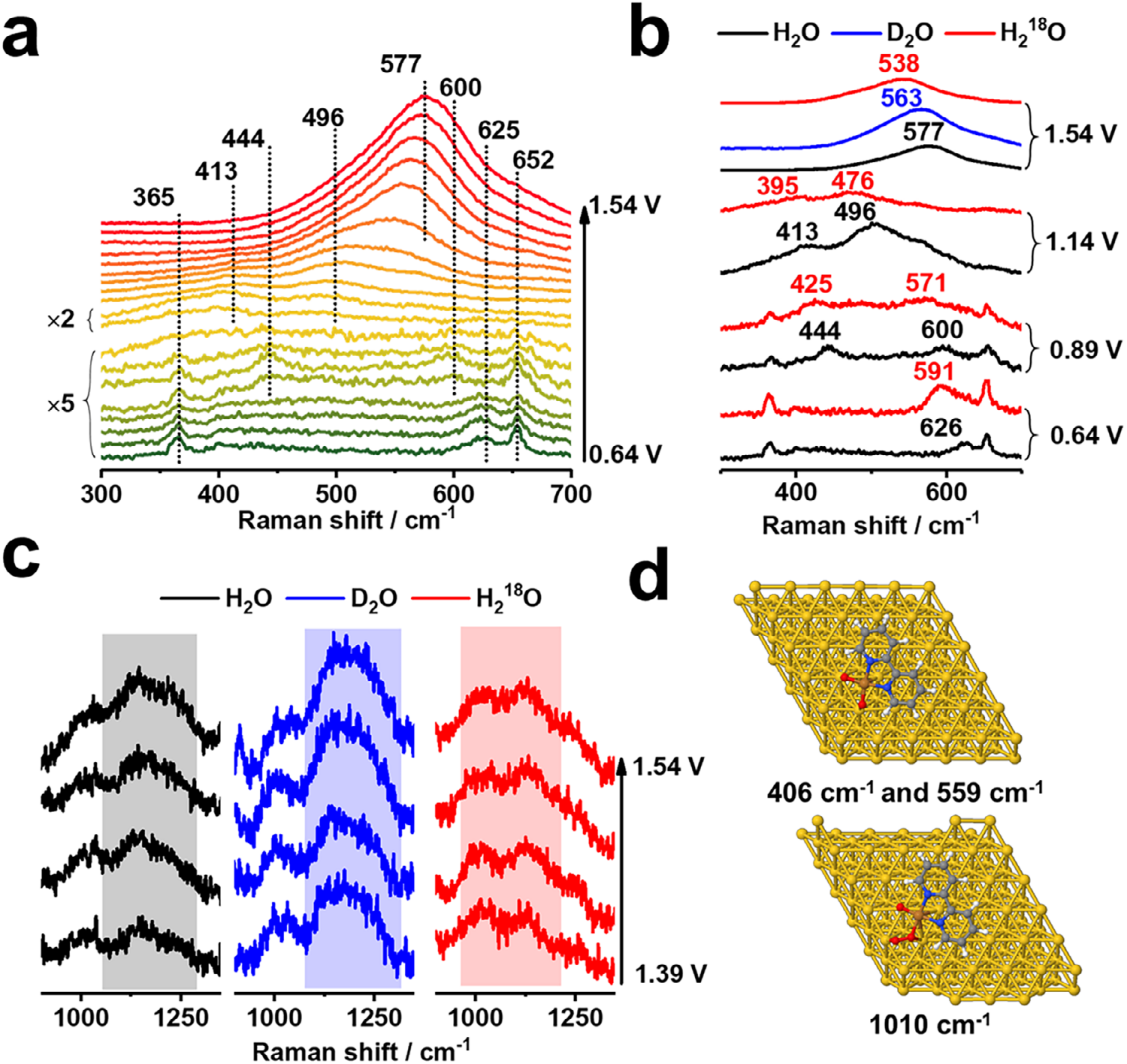
(a) In situ electrochemical Raman spectra of (bpy)Cu(OH)_2_/Au from 0.64 to 1.54 V vs RHE. (b) Isotopic Raman experiments of (bpy)Cu(OH)_2_/Au in the frequency range between 300 and 700 cm^−1^. (c) In situ electrochemical Raman spectra of (bpy)Cu(OH)_2_/Au from 1.39 to 1.54 V vs RHE at 1100–1300 cm^−1^. (d) Optimized adsorption configurations and vibration frequencies of Cu(III)‐O‐Au and Cu(III)‐OO‐Au. Atom keys: Cu (brown), Au (yellow), C (gray), N (blue), O (red), and H (white).

**Figure** **2**a shows the potential‐dependent Raman spectra acquired at (bpy)Cu(OH)_2_/Au from 0.64 to 1.54 V with 0.05 V intervals. A further increase in potential could drive intense OER to generate bubbles that hinder the collection of Raman signals. The reversibility of SHINERS spectra could be found in Figure S7 (Supporting Information). The 200–1800 cm^−1^ spectral range contains all the most characteristic peaks of (bpy)Cu(OH)_2_ on Au (Figure S8, Supporting Information). We focus on low‐wavenumber regions containing metal‐molecular interactions, surface species, and important intermediates. From the initial potential of 0.64 to 1.04 V, a distinct band at 625 cm^−1^, which can be assigned to the stretching vibration of Cu(II)‐O (*v*_Cu(II)‐O_),^[^ ^14a^ ^]^ becomes more intense, reaches a maximum at 0.79 V, and then becomes weaker with increasing potential. A similar band was also observed for the oxidation of Cu(OH)_2_/Au, as shown in Figure S9 (Supporting Information). Furthermore, the results of the isotopic experiments in Figure 2b clearly show that the band at 625 cm^−1^ redshifts by 5.4% in ^18^O water but does not shift in deuterated ^16^O water. These results prove that the initial (bpy)Cu(OH)_2_ is oxidated and deprotonated to (bpy)Cu(II)O_2_ on the Au electrode.

At 0.84 V, *v*_Cu(II)‐O_ at 625 cm^−1^ disappears, whereas two new Raman bands appear at 444 and 600 cm^−1^ and become stronger and reach a maximum at 0.94 V as the potential increases. In addition, these two new Raman bands also shift to 425 and 571 cm^−1^ in ^18^O water but remain unchanged in deuterated ^16^O water (Figure 2b). Previous Raman results revealed the Cu–O stretching vibration of a Cu(III) oxide at 603 cm^−1^,^[^ ^14a^ ^]^ which is very close to our observations. Thus, we attributed these two bands to the Cu(III)‐O stretching vibration (*v*_Cu(III)‐O_). This direct molecular evidence further proves that the broad anodic peak in LSV also consists of the oxidation of Cu(II) to Cu(III).

**Figure 3:**
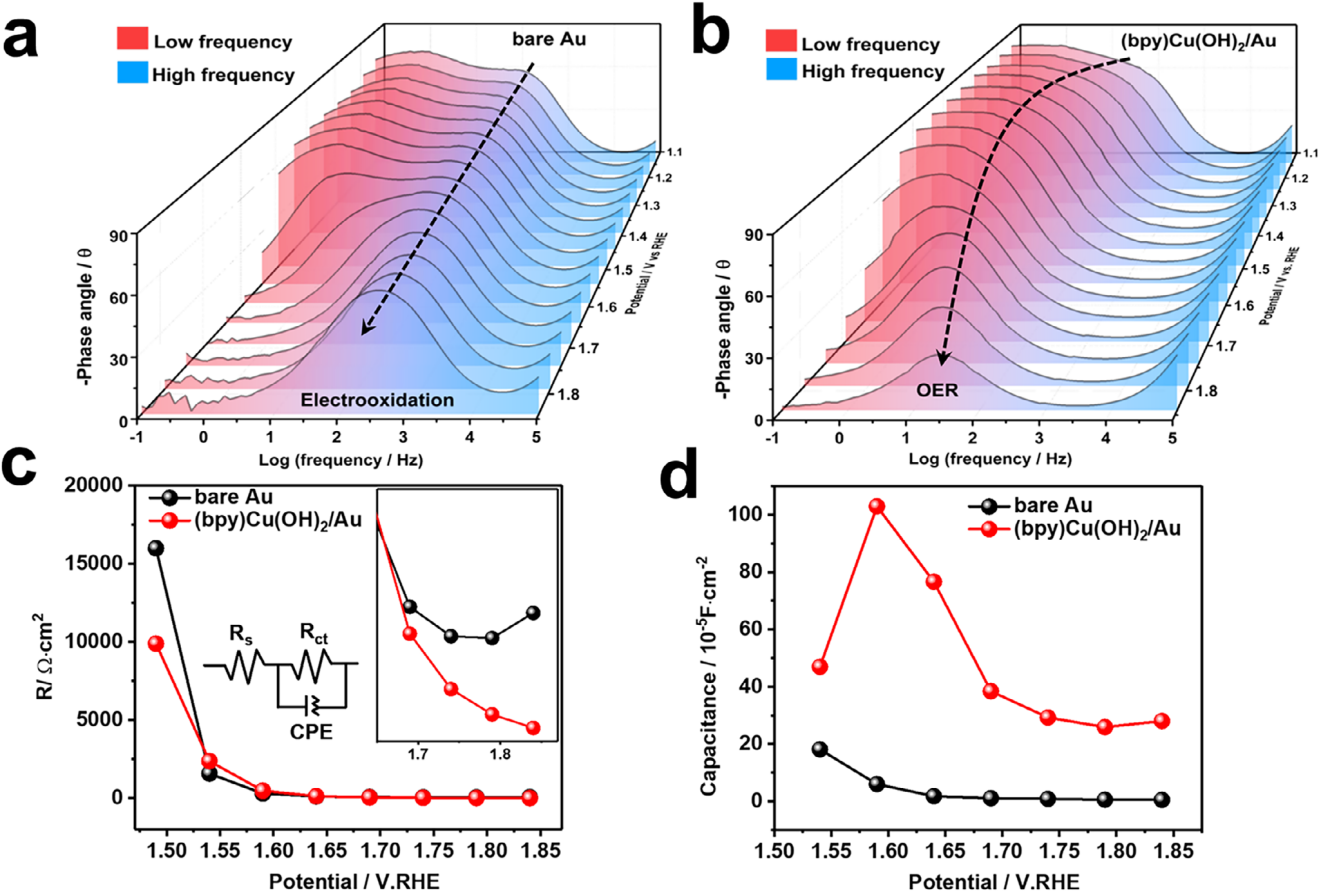
In situ electrochemical Bode phase plots of (a) bare/Au and (b) (bpy)Cu(OH)_2_/Au. (c) R_ct_ values of the Au electrode at different applied potentials in different electrolytes were obtained from the EIS fitting data. (d) Double‐layer capacitance, C_dl_, $C_{\mathrm{dl}}=\frac{{\left(\mathrm{CPE}\cdot R_{\mathrm{ct}}\right)}^{1/n}}{R_{\mathrm{ct}}}$, the relevant parameter values were fitted by Zview2 software.

As the potential increased to 1.04 V, the two bands attributed to *v*_Cu(III)‐O_ shifted to 413 and 496 cm^−1^. The 496 cm^−1^ band then evolves into an intense and broad band at 500–620 cm^−1^, which is typically assigned to the Au–O vibration. Furthermore, density functional theory (DFT) calculations also revealed that the bands at ≈406 and 559 cm^−1^ could be assigned to Cu(III)‐O‐Au vibrations (*v*_Cu(III)‐O‐Au_) (Figure 2d). Moreover, these two bands at 413 and 496 cm^−1^ also show redshifts of 5.2 % and 6.2 % in ^18^O water, in contrast to the lack of a shift in deuterated ^16^O water (**Figure** **3**b). These results indicate that the electrochemical potential oxidizes Cu(II)‐O into Cu(III)‐O, which bridges the Au surface to form (bpy)Cu(III)O_2_‐Au with interfacial oxygen‐bridged binuclear metal centers of Cu(III)‐O‐Au.

When the potential is above 1.24 V, a new broad band appears at 1100–1300 cm^−1^ (Figure 2c). Typically, many Raman experiments have proven that this band is due to the O‐O stretching of *O_2_ intermediates for the OER.^[^ ^16^ ^]^ The ^18^O isotope substitution experiments with a redshifted frequency also confirmed that these bands were related to O‐O stretching. In addition, the DFT simulation of Cu(III)‐OO‐Au has a vibration frequency at 1010 cm^−1^ (Figure 2d). Meanwhile, the intense band at 577 cm^−1^ also redshifts by 2.1 % in ^18^O water and 6.2 % in deuterated ^16^O water (Figure 2b). This can be attributed to the Au‐O stretching with a large amount of *OH adsorbed on the Au oxide surface (denoted as *v*_Au‐O/OH_).^[^ ^18^ ^]^ Therefore, the interfacial oxygen‐bridged binuclear metal centers of Cu(III)‐O‐Au might combine with surface *OH to form Cu(III)‐OOH‐Au, then turn into Cu(III)‐OO‐Au to release O_2._ This kind of O‐O bond formation pathways are also proposed in dinuclear molecular catalysts.^[^ ^7^, ^19^ ^]^ When the potential further increase above 1.34 V, the Raman signals of molecular catalyst become weaker and disappear, which might arise from the molecules could desorb or do not adsorb on the surface in a stable configuration at these high potentials. But they could participate in the reaction process dynamically, which is supported by cycling the controlled potential electrolysis in the solutions with or without molecular catalysts (Figure S10, Supporting Information).

To further explore the reaction kinetics of interfacial oxygen‐bridged binuclear metal centers, we employed operando EIS to. Study the interface behavior during the OER. The peak frequency of the Bode plots and corresponding Nyquist plots was used to reveal the interfacial charge transfer behavior in different phases.^[^ ^20^ ^]^ Figure 3a,b shows the Bode phase plots from 1.14 to 1.84 V. For (bpy)Cu(OH)_2_/Au, there is always an obvious peak located in the low‐frequency region (below 10^2^ Hz), in contrast to other intense peaks located in the high‐frequency region (above 10^2^ Hz) for bare Au. Typically, the low‐frequency peak is associated with the oxidation of adsorbed species or the OER, whereas the high‐frequency peak is attributed to the oxidation reaction of catalysts.^[^ ^20a,c^ ^]^ Therefore, the absence of these peaks in the high‐frequency region for (bpy)Cu(OH)_2_/Au further suggests that the interfacial molecular catalysts prevent the Au electrode from oxidizing into a high‐valence state (e.g., AuOOH), which is in line with the results of LSV.

In addition, from 1.39 to 1.54 V, the characteristic peak of (bpy)Cu(OH)_2_/Au transforms to a new peak in the 10^1^−10^2^ Hz frequency region, corresponding to the beginning of the OER according to previous reports.^[^ ^20c^ ^]^ The control samples of bare Au, only display intense peaks in the 10^2^−10^3^ Hz frequency region. Moreover, the phase angle in the Bode plot first slightly decreases and then rapidly decreases above 1.54 V for (bpy)Cu(OH)_2_/Au (Figure 3b). This indicates that the catalyst surfaces become more conductive for the adsorption of reaction intermediates during the OER process.^[^ ^21^ ^]^

The Nyquist plots are further used to fit the equivalent resistances via an equivalent circuit, which consists of the electrolyte resistance (R_s_), charge‐transfer resistance (R_ct_), and constant phase element (CPE) that emulates the double‐layer capacitance (DLC, C_dl_). As shown in Figure 3c, the potential‐dependent R_ct_ rapidly decreases at ≈1.54 V when the OER occurs. This is consistent with the LSV and phase angle variation. (bpy)Cu(OH)_2_/Au always has the smallest R_ct_, especially when the potential further increases to drive violent OER occurrence (Figure 3c inset). This proves that the fastest charge transfer rate occurred during the OER with the (bpy)Cu(OH)_2_/Au catalyst.

Furthermore, (bpy)Cu(OH)_2_/Au also has the largest C_dl_ values at potentials above 1.54 V compared with those of the other control samples (Figure 3d). In line with previous reports,^[^ ^20^, ^21^ ^]^ the large capacitive behavior corresponds to a large amount of charge accumulation from the adsorption of hydroxyl ions. Thus, these results prove that the molecular catalyst on the Au electrode has larger charge accumulation, leading to lower resistance and fast mass transport of OH^‐^ to promote the OER.

**Figure 4:**
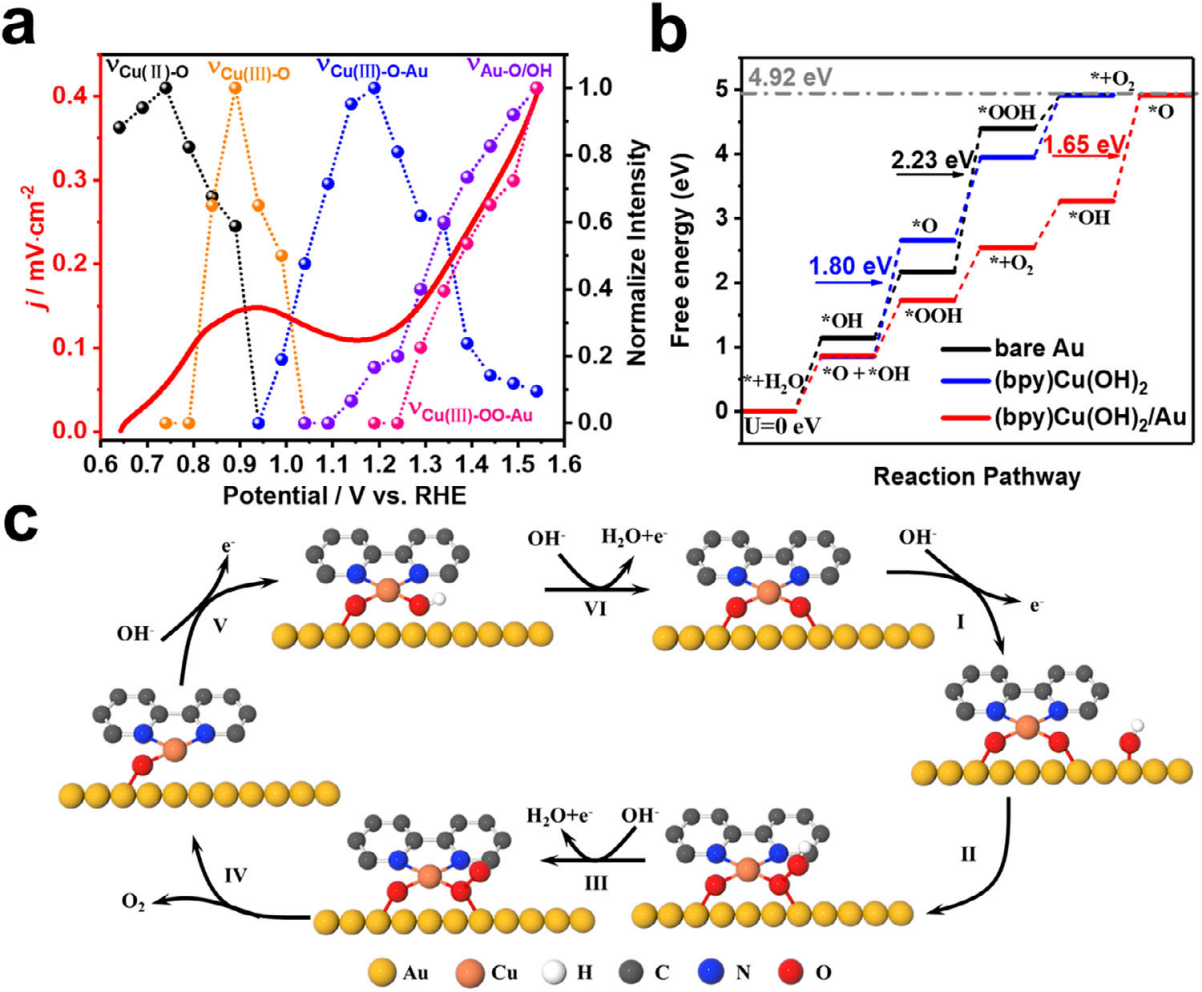
(a) Normalized Raman intensity of *v*_Cu(II)‐O_, *v*_Cu(III)‐O_, *v*_Cu(III)‐O‐Au_, *v*_Au‐O/OH_, and *v*_Cu(III)‐OO‐Au_ corresponding to the LSV. (b) Free energy for the OER on bare Au, (bpy)Cu(OH)_2_, and (bpy)Cu(OH)_2_/Au. (c) OER mechanism with optimal conformations of the (bpy)Cu(OH)_2_/Au model.

To clearly correlate the observed intermediates with the reaction mechanism, the normalized band intensity variations of *v*_Cu(II)‐O_ at 625 cm^−1^, *v*_Cu(III)‐O_ at 600 cm^−1^, *v*_Cu(III)‐O‐Au_ at 496 cm^−1^, and *v*_Au‐O/OH_ at 577 cm^−1^ are compared with the LSV of (bpy)Cu(OH)_2_/Au in **Figure** **4**a. From 0.64 to 0.94 V, the intensity of *v*_Cu(II)‐O_ clearly decreases, whereas the intensity of *v*_Cu(III)‐O_ increases to the maximum. This indicates that the broad anodic peak at 0.84 V in LSV is attributed to the oxidation of Cu(II) to Cu(III). As the potential further increases, *v*_Cu(III)‐O_ turns into *v*_Cu(III)‐O‐Au_. Then, *v*_Au‐O/OH_ appears and combines with *v*_Cu(III)‐O‐Au_ to form *v*_Cu(III)‐OOH‐Au_, which quickly combines with *OH and form *v*_Cu(III)‐OO‐Au_ to release O_2_. Thus, the *j* value of the OER increases with increasing Raman band intensity of *v*_Au‐O/OH_ and *v*_Cu(III)‐OO‐Au_. Combined with the EIS results, the formation of an interfacial oxygen‐bridged binuclear metal center effectively promoted the conversion of *OH to O_2_ to improve water electrolysis.

Based on the directly observed molecular evidence, Gibbs free energy calculations were applied to further investigate the reaction mechanism of the OER. In line with the potential‐ dependent Raman results, dependent Raman results, (bpy)Cu(OH)_2_ was adsorbed onto the Au(111) surface and oxidized into (bpy)Cu(III)O_2_‐Au with oxygen‐bridged binuclear metal Centers of Cu(III)‐O‐Au for the OER. The subsequent steps are displayed in Figure 4b,c. The surface *OH on the Au electrode combines with Cu(III)‐O‐Au to generate the *OOH intermediate (Cu(III)‐OOH‐Au), which requires an additional energy input of 0.85 eV. However, the direct combination of Cu(III)‐O‐Au with OH from solution requires an additional energy input of 1.71 eV (Figure S11, Supporting Information). These findings are in line with the Raman observation that *v*_Au‐O/OH_ combines with *v*_Cu(III)‐O‐Au_ to start the OER. Further dehydrogenation of *OOH leads to the formation of *O_2_, with a Δ*G* value of 0.72 eV. Oxygen molecules then desorb easily from the surface, requiring only 0.11 eV. Next, the Cu atom remains with one oxygen atom bonded and combines with another hydroxide group, with a Δ*G* value of 0.72 eV. As discussed above, the dehydrogenation of the OH group on Cu(III)‐OOH‐Au is identified as the potential‐determining step (PDS) in this process, with an overpotential of 0.42 V.

Comparative calculations were performed for the OER process on pure Au and pure (bpy)Cu(OH)_2_ catalysts in Figure 4b. The results revealed that the PDS in the OER process on pure (bpy)Cu(OH)_2_ involves the formation of *O, resulting in an overpotential of 0.57 V. In contrast, on a pure Au catalyst, the PDS involved the dehydrogenation of *O and water molecules, leading to *OOH, with a significantly higher overpotential of 1.0 V. These overpotential values are much greater than those observed for the catalyst in this work. Therefore, the synergistic combination of gold and (bpy)Cu(OH)_2_ in this study has been shown to modify the elementary reaction steps and PDS of the OER, consequently effectively reducing the reaction overpotential. From the above discussion, the reaction pathway and mechanism of OER for (bpy)Cu(OH)_2_/Au catalyst are schematically illustrated in Figure 4c. The interfacial oxygen‐bridged binuclear metal centers of Cu(III)‐O‐Au promote the conversion of *OH into O_2_, which is in line with the results of the LSV and Raman experiments.

## 3 Conclusion

In summary, in situ shell‐isolated nanoparticle‐enhanced Raman spectroscopy and electrochemical impedance spectroscopy have been successfully used to probe the dynamic process, interfacial structure, and intermediates of a heterogenized molecular catalyst of (bpy)Cu(OH)_2_/Au during water electrolysis. Linear‐sweep voltammetry indicates a 14‐fold enhancement in OER activity over the heterogenized molecular catalyst at the overpotential of 0.4 V. Direct Raman molecular evidence and theoretical calculations suggest that the reaction pathway is as follows: the interfacial (bpy)Cu(OH)_2_ first oxidizes into Cu(III) and bridges to Au atoms via oxygenated species, such as the formation of (bpy)Cu(III)O_2_‐Au with the oxygen‐bridged binuclear metal centers of Cu(III)‐O‐Au as active sites. As the potential further increases, Cu(III)‐O‐Au combines with hydroxyl groups to form the intermediate of Cu(III)‐OOH‐Au and then transforms into Cu(III)‐OO‐Au to release O_2_. Furthermore, in situ EIS supports that the interfacial Cu(III)‐O‐Au has low resistance and fast mass transport of OH^‐^. Gibbs free energy calculations also prove that the formation of Cu(III)‐O‐Au significantly modifies the elementary reaction steps and lowers the overpotential for the OER. This work offers theoretical and experimental support for the advancement and industrial implementation of the OER by using heterogeneous molecular catalysts.

## 4 Experimental Section

### Preparation of SHINs

In this paper, a seed‐mediated growth method^[^ ^22^ ^]^ was used to prepare the 120 nm Au nanoparticles (NPs) as follows: 1.5 mL of 1 wt.% sodium citrate solution was added into 50 mL of 0.01 wt.% boiling HAuCl_4_ solution with stirring to obtain 16 nm Au seeds. Then, to obtain 50 nm Au NPs, 1.5 mL of the as‐prepared 16 nm Au seeds, 300 µL of 1 wt.% ascorbic acid, 100 µL of 1 wt.% sodium citrate solution, and 10 mL of Milli‐Q water were added into a flask in the ice‐water bath. After stirring for 5 min, 550 µL of 1 wt.% HAuCl_4_ solution was dropwise added into the mixture and then moved to 70 °C bath for 30 min. Next, 2 mL of the as‐prepared 50 nm Au seeds, 400 µL of 1wt.% ascorbic acid, 100 µL of 1 wt.% sodium citrate solution, and 20 mL of Milli‐Q water were added to a flask with stirring in the ice‐water bath. After 5 min, 654 µL of 1 wt.% HAuCl_4_ was dropwise added into the mixture and moved to a 70 °C bath for 30 min, and 120 nm Au NPs were obtained.

The SHINs were synthesized according to the previous reports.^[^ ^9a^ ^]^ First, 6 mL of the as‐prepared 120 nm Au core solution was added into a flask with stirring. After the solution was stirred for 10 min, 0.2 mL of 1mM APTMS solution was added and stirred for 15 min. Next, 3.2 mL of 0.54 wt.% sodium silicate solution was added into the mixture and stirred overnight at room temperature. A thin pinhole‐free silica shell was coated to the Au core. Finally, the solution was centrifuged at 3000 rpm/min for 10 min and washed with Milli‐Q water twice for later use.

### Preparation of ((bpy)Cu(OH)_2_) Complex

The preparation procedures of ((bpy)Cu(OH)_2_) complex are as follows: sequentially mixing 1 mL of 10 mm 2,2'‐bipyridine (BPY) aqueous solution, 1 mL of 10 mm copper acetate (Cu(OAc)₂) aqueous solution, 0.5 mL of 2 m sodium acetate (NaOAc) buffer solution, and 7 mL of deionized water (18.2 MΩ·cm) under ultrasonication. Then, 0.5 mL of 2 m sodium hydroxide solution was added dropwise and ultrasonicated for 5 min to obtain a blue solution.

## Conflict of Interest

The authors declare no conflict of interest.

## Supporting information



Supporting Information

Supporting Information

## Data Availability

The data that support the findings of this study are available from the corresponding author upon reasonable request.
