# Supplementary material for: Uncovering Interfacial Oxygen‐Bridged Binuclear Metal Centers of Heterogenized Molecular Catalyst for Water Electrolysis
Source: Adv Sci (Weinh). 2025 Mar 30;12(22):2417607. doi: 10.1002/advs.202417607 (PMC12165092; doi:10.1002/advs.202417607)
Supplement: Supplementary file 1 — Supporting Information [file ADVS-12-2417607-s001.docx]

Uncovering Interfacial Oxygen-Bridged Binuclear Metal Centres of Heterogenized Molecular Catalyst for Water Electrolysis

Zhou Yu,^#,[a]^ Jian-Ping Li,^#,[a]^ Xian-Kun Xu,^[a]^ Zhong-Chen Ding,^[a]^ Xiao-Hui Peng,^[a]^ Yi-Jing Gao,^*,[a,b]^ Qiang Wan,^[a]^ Ju-Fang Zheng,^[a]^ Xiao-Shun Zhou,*^,[a]^ Ya-Hao Wang*^,[a]^

[a] Z. Yu, J.-P. Li, X.-K. Xu, Z.-C. Ding, X.-H. Peng, Y.-J. Gao, Q. Wan, J.-F. Zheng, X.-S. Zhou, and Y.-H. Wang
Key Laboratory of the Ministry of Education for Advanced Catalysis Materials, Institute of Physical Chemistry, College of Chemistry and Materials Science,
Zhejiang Normal University
Jinhua 321004, P. R. China
E-mail: [xszhou@zjnu.edu.cn](mailto:xszhou@zjnu.edu.cn); yahaowang@zjnu.edu.cn
# These authors contributed equally

[b] Y.-J. Gao
Zhejiang Engineering Laboratory for Green Syntheses and Applications of Fluorine-Containing Specialty Chemicals, Institute of Advanced Fluorine-Containing Materials,
Zhejiang Normal University,
Jinhua 321004, P. R. China
E-mail: yijinggao@zjnu.edu.cn

# Experimental Section

**Electrochemical measurements.**

Electrochemical measurements were carried out with a CHI-600D potentiostat using a custom three-compartment glass cell. A carbon rod and Ag/AgCl were used as the counter and reference electrodes, respectively. The solutions used for the electrochemical measurements were deaerated and protected by argon. In situ electrochemical impedance spectroscopy (EIS) tests were performed over a frequency range from 10^5^–10^–1^ Hz. The equivalent circuit Rs (CPE Rct) is used for fitting the data, where Rs is the solution resistance, CPE is the constant phase element and Rct is the charge transfer resistance. The relevant parameter values were fitted via ZView2 software.

**In situ Raman measurements.**

Raman experiments were carried out on a confocal microscope Raman system (Renishaw InVia). The excitation wavelength was 632.8 nm, and a 50× microscope objective with a numerical aperture of 0.55 was used in all Raman measurements. The as-prepared 120 nm Au @ ca. 2 nm SiO_2_ nanoparticles were dropped on the Au(111) electrode as Raman signal amplifiers. A homemade Raman cell with potential control at a CHI660E potentiostat was used for in situ electrochemical Raman experiments.

**Computational methods.**

The Vienna ab initio simulation package (VASP) was used with the Perdew–Burke–Ernzerhof (PBE) functional of the generalized gradient approximation (GGA) for density functional theory (DFT) calculations to model the electron exchange correlation energy.^[3, 4]^ The projector augmented wave (PAW) method^[5]^ was utilized to capture electron‒ion interactions, with a cut-off energy of 450 eV. For the Au(111) surface slab models, a 3 × 3 supercell consisting of three atomic layers, in which the bottom two atomic layers were fixed, was used. The K-point^[6]^ was set as 2 × 2 × 1. For pure (bpy)Cu(OH)_2_, a simulation cell dimension of 20 × 20 × 20 Å³ was applied with a K-point mesh of size 1 × 1 × 1. For (bpy)Cu(OH)_2_/Au, the K point was set as 2 × 2 × 1. Convergence criteria were set at 1 × 10^-5^ eV for energy and -0.01 eV/Å for force. van der Waals (vdW) interactions were accounted for via the DFT-D3 method.^[7]^ A 15 Å vacuum layer along the z direction was introduced to prevent interlayer interactions.

Utilizing the computational hydrogen electrode (CHE) model proposed by Nørskov et al.,^[8, 9]^ the free energy change (Δ*G*) for each elementary step was determined as follows:

Δ*G* = Δ*E* + Δ*E*_ZPE_ - *T*Δ*S* + Δ*G*_U_ + Δ*G*_pH_

Here, Δ*E* signifies the total energy computed through DFT, Δ*E*_ZPE_ is the zero-point energy (ZPE) correction derived from vibrational frequency calculations, TΔ*S* represents the entropy contribution at T = 298.15 K, and Δ*G*_U_ represents the electrode potential (*U*) contribution, which can be calculated as Δ*G*_U_ = -*n*e^-^*U*. Additionally, Δ*G*_pH_ denotes the pH correction for free energy and can be determined by Δ*G*_pH_ = -k_B_Tln[H^+^] = k_B_T × ln10 × pH, where k_B_ represents the Boltzmann constant. Finally, in the Δ*G* calculation, the free energy of H^+^ + e^-^ is considered equivalent to 1/2H_2_.

**Figures**


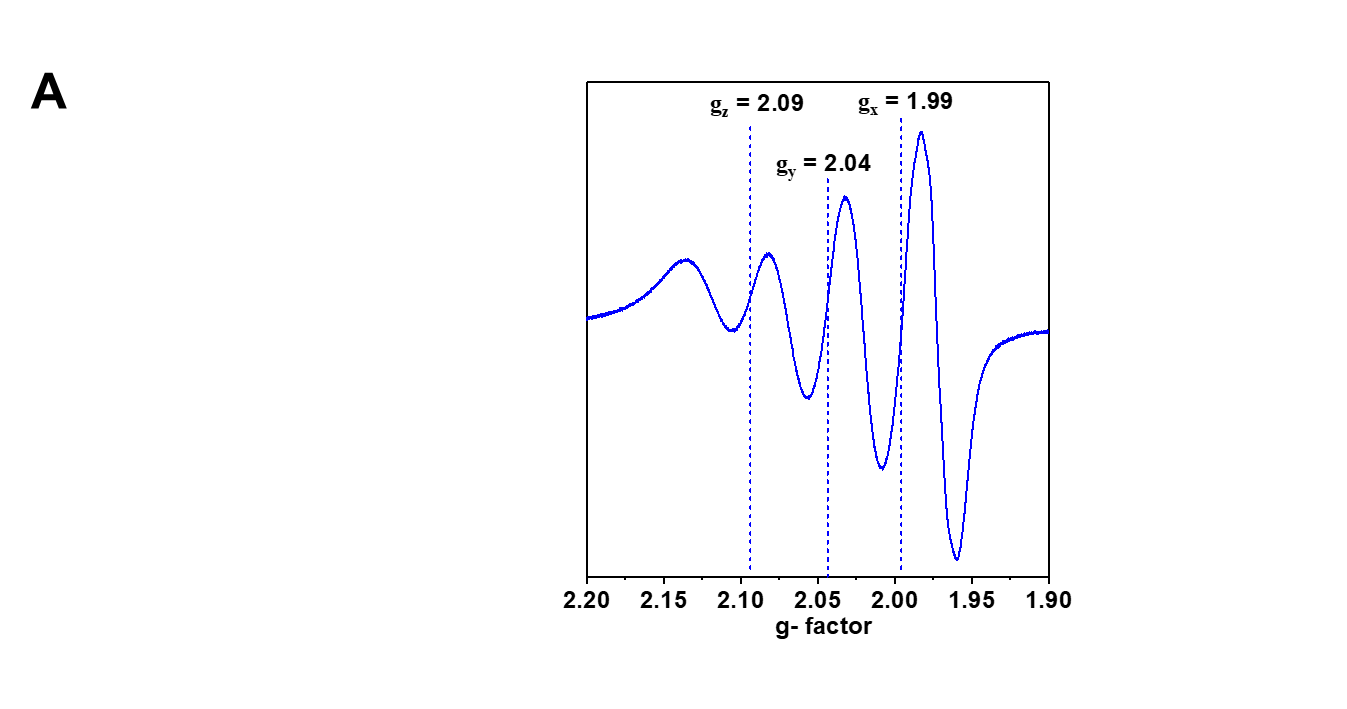


**Figure S1** The g-value of the (bpy)Cu(OH)_2_ aqueous solution ( pH=12.5).


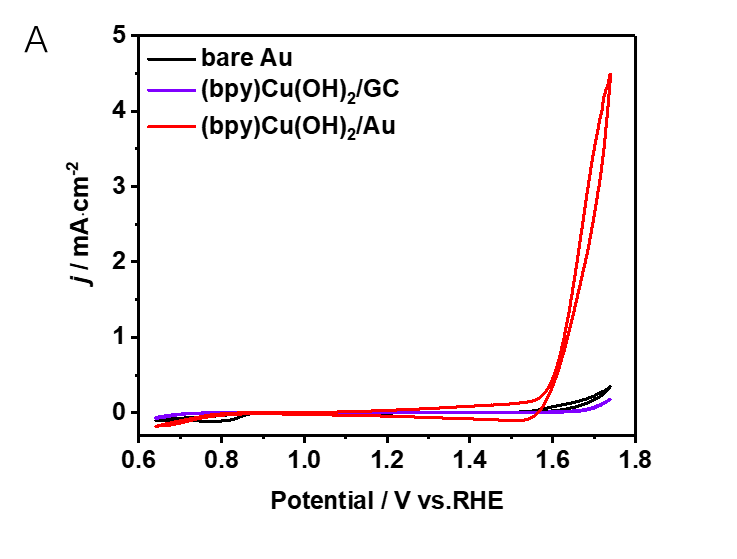


**Figure S2** CVs of the OER on the bare Au electrode (black), (bpy)Cu(OH)_2_/GC electrode (purple) and (bpy)Cu(OH)_2_/Au electrode (red) at pH=12.5. The scan rate was 1 mV s^-1^.

**
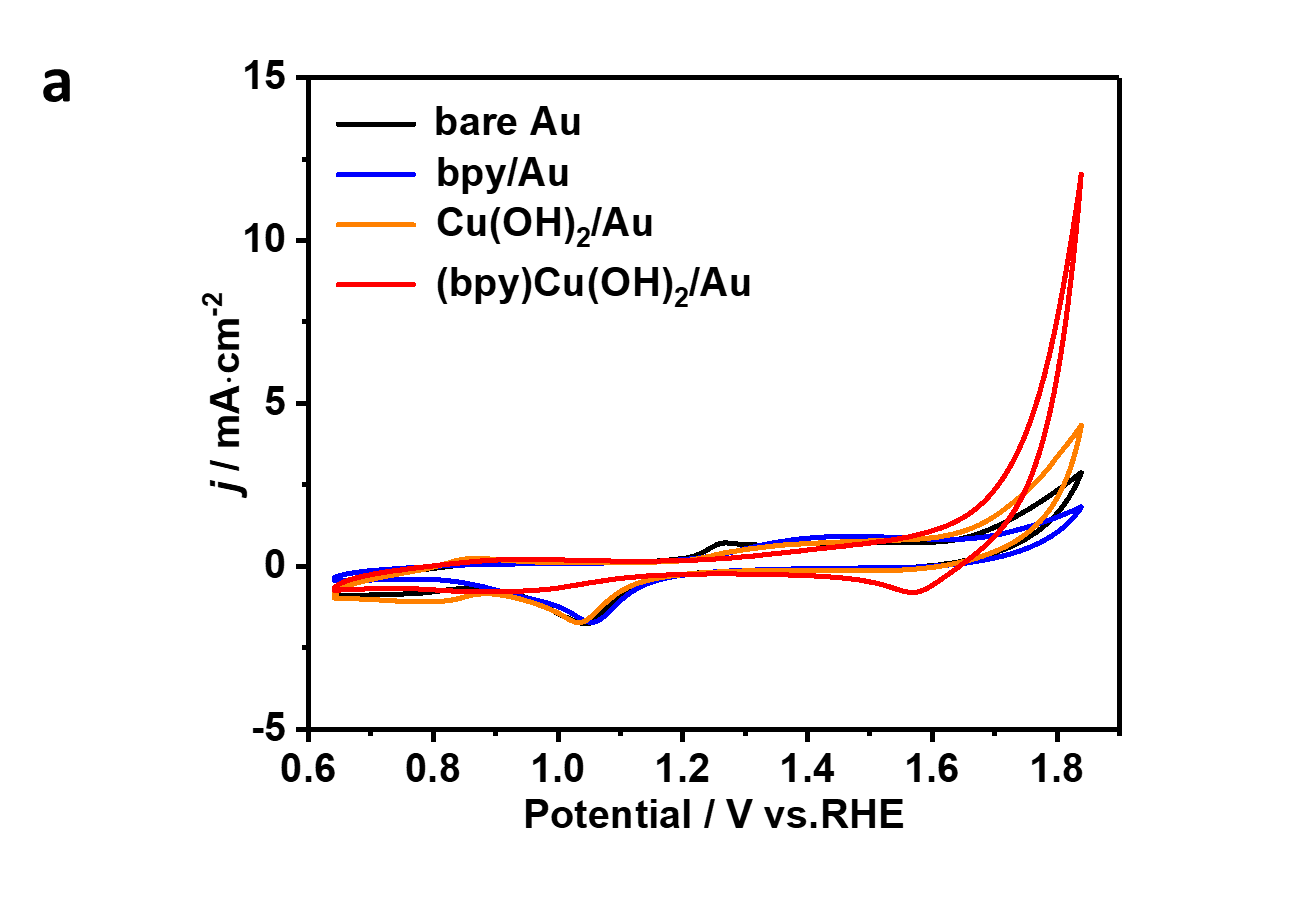
**

**Figure S3** The CVs of OER on the bare Au electrode (black), bpy/Au electrode (blue), Cu(OH)_2_/Au (orange) and (bpy)Cu(OH)_2_/Au (red) in pH=12.5, respectively. Scan rate is 100 mV s^-1^.


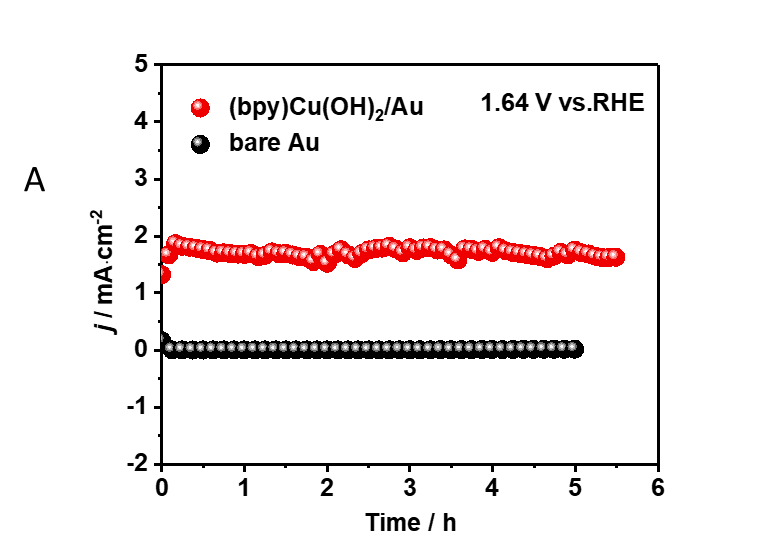


**Figure S4** The I-t curve of the (bpy)Cu(OH)₂/Au electrode and bare Au electrode were recorded under a constant applied potential of 1.64 V vs. RHE.


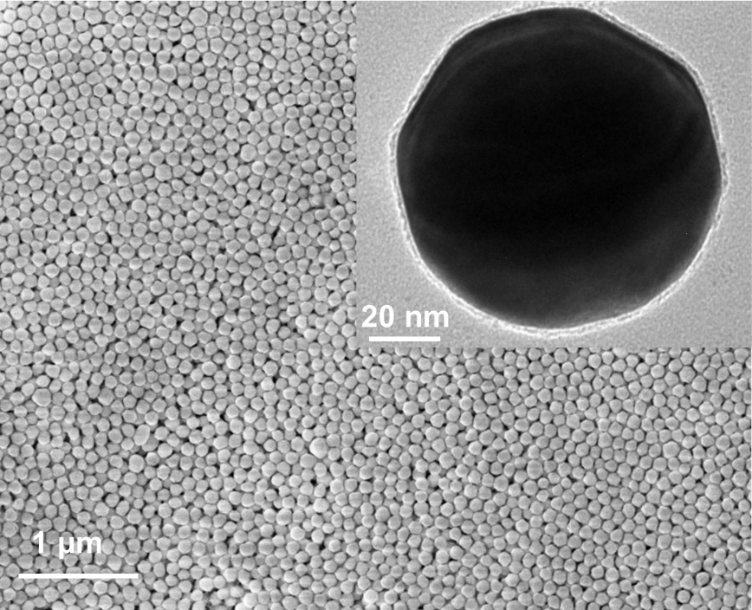


**Figure S5** SEM image of shell-isolated nanoparticles (SHINs). Typical TEM image of an individual SHIN.


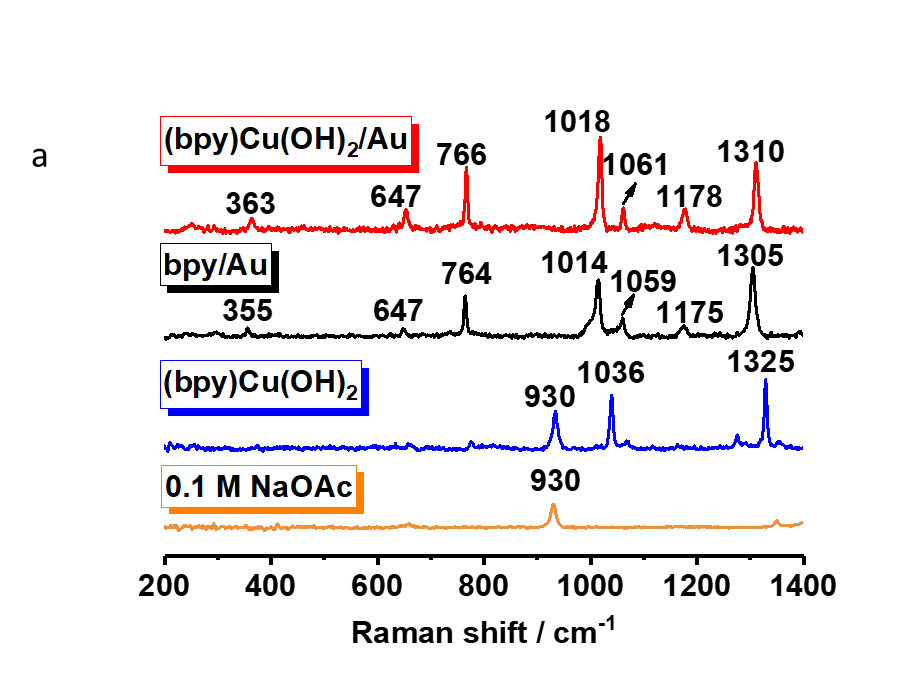


**Figure S6** The SHINERS spectra of bpy/Au, (bpy)Cu(OH)_2_/Au compared to the normal Raman spectra of 8 mM (bpy)Cu(OH)₂ and 0.1 M NaOAc electrolyte in solution.


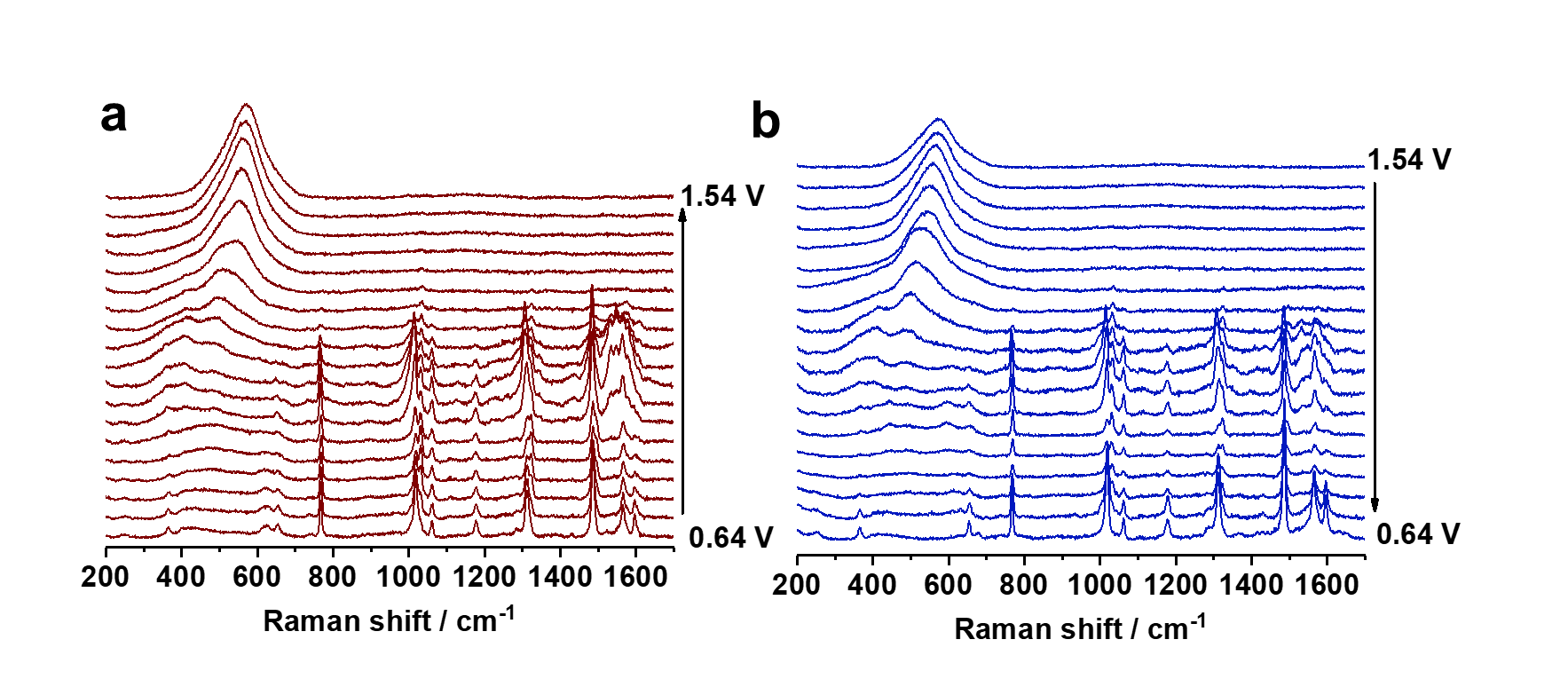


**Figure S7** In situ Raman spectra of (bpy)Cu(OH)₂/Au electrode recorded under (a) forward and (b) reverse potential scans with a potential interval of 0.05 V.The 200–1800 cm^−1^ spectral range contains all the most characterize peaks of (bpy)Cu(OH)_2_ on Au.

**
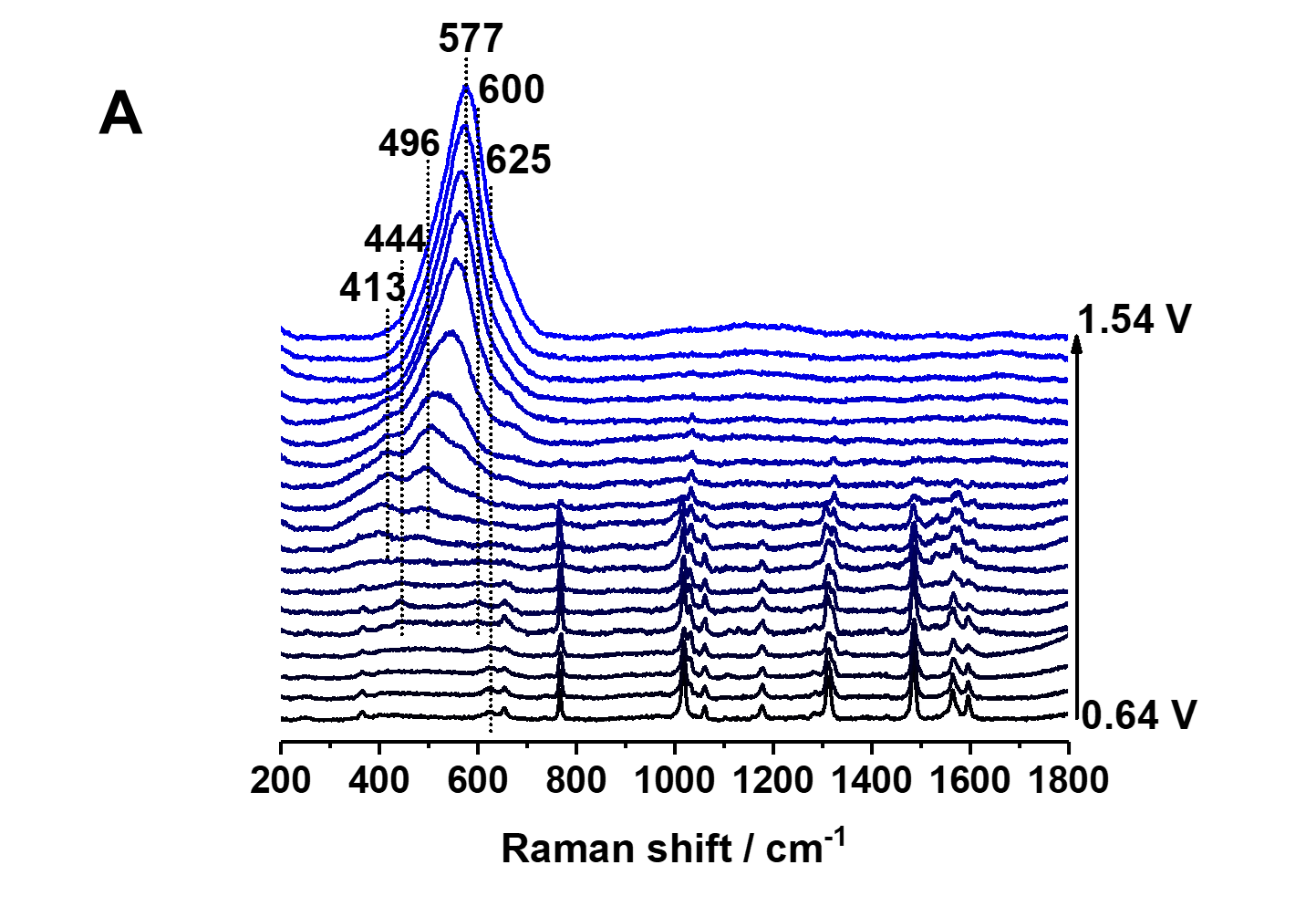
**

**Figure S8** The 200–1800 cm^−1^ spectral range contains all the most characterize peaks of (bpy)Cu(OH)_2_ on Au.

**
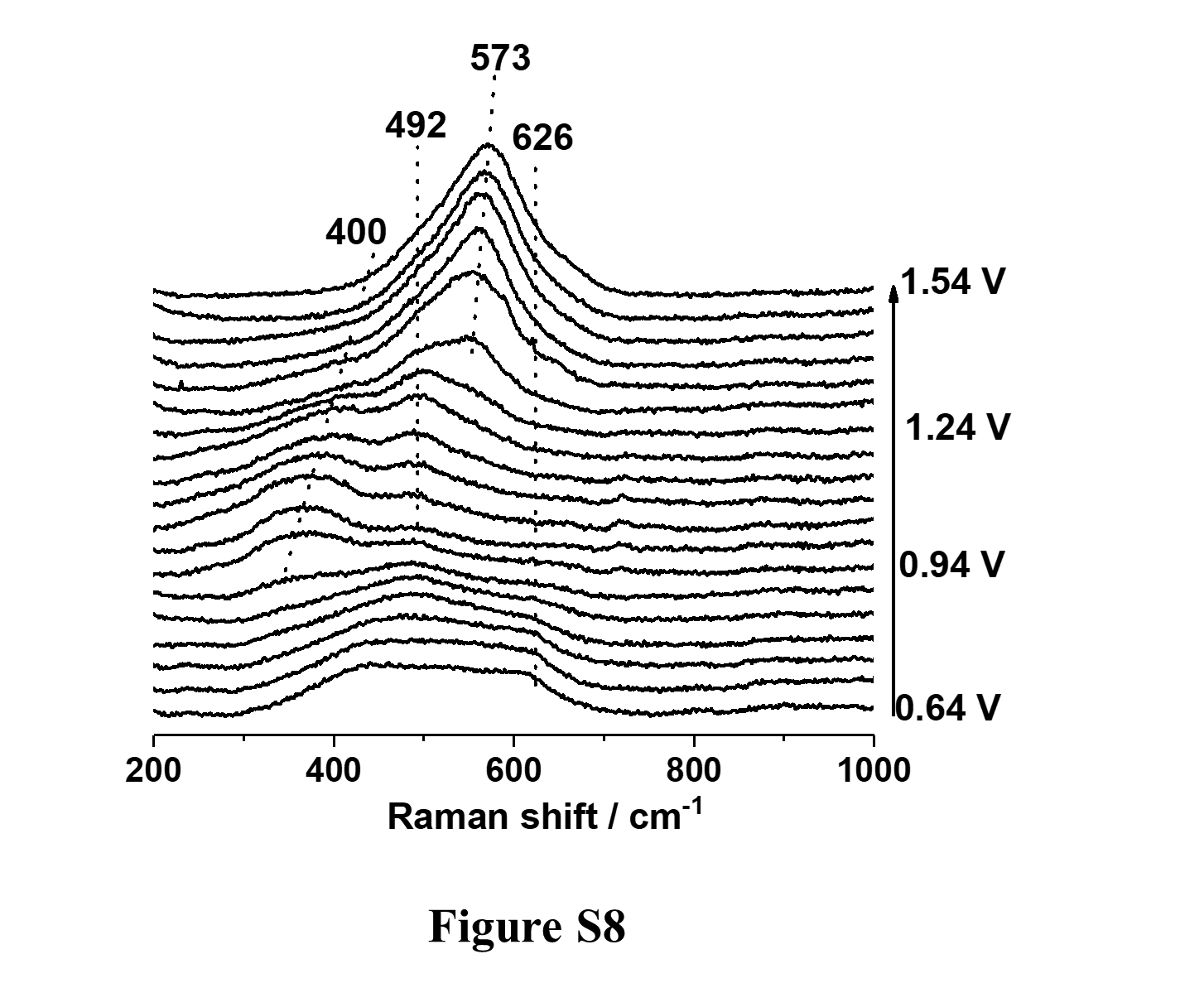
**

**Figure S9** In situ electrochemical Raman spectra of the Cu(OH)_2_/Au at 0.64-1.54 V (vs. RHE).


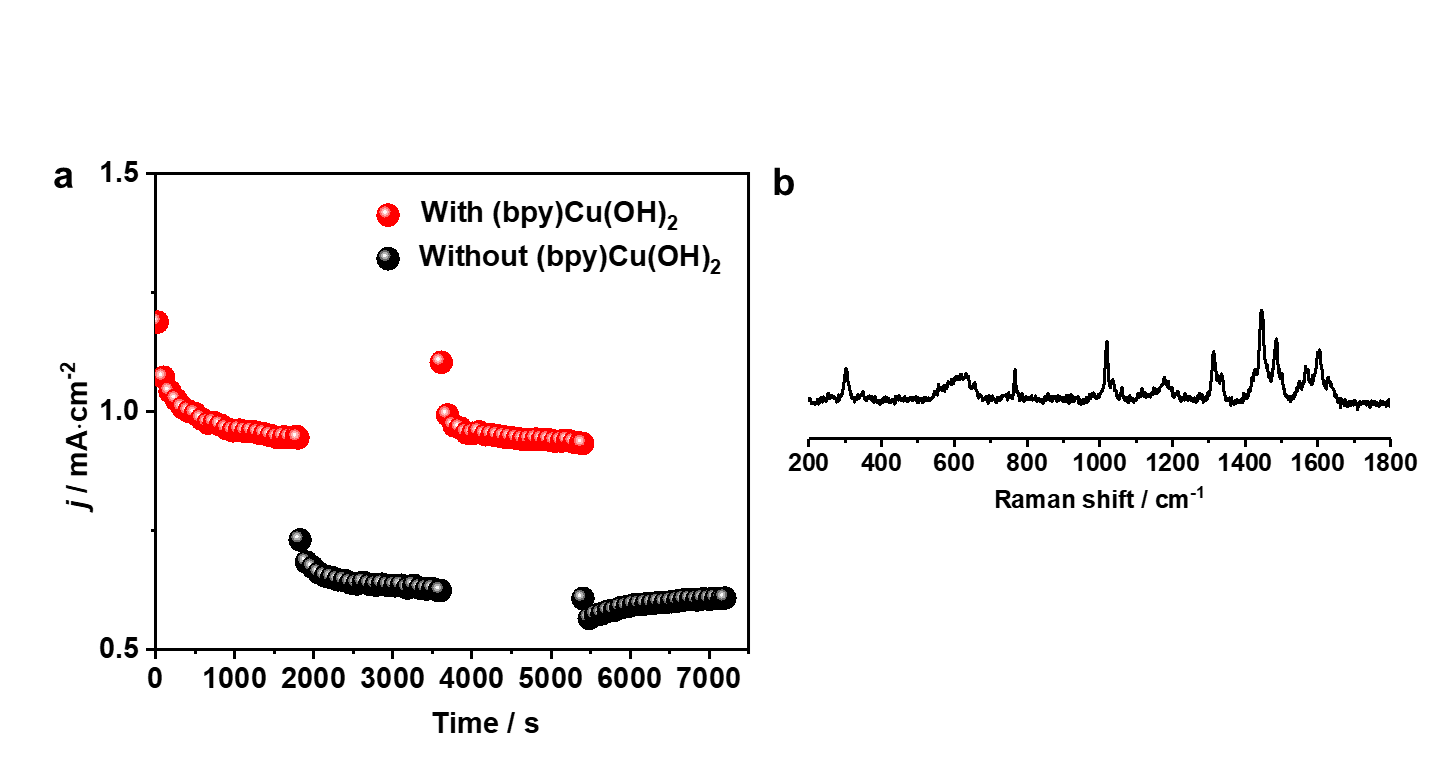


**Figure S10** (a)The I-t curve of Au electrode cycling operated at 1.64 V vs. RHE in the solutions with or without 1 mM (bpy)Cu(OH)₂/Au molecules (b) The SHINERS spectra of Au electrode obatined in air after the two cycles of controlled potential electrolysis at 1.64 V.

**
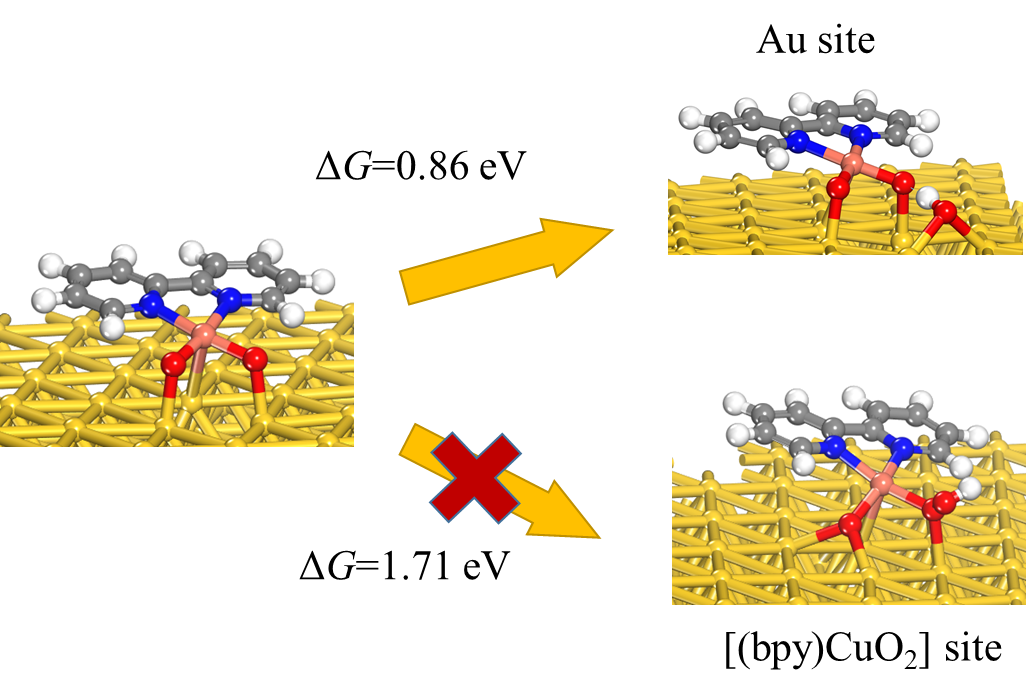
**

**Figure S11** Gibbs free energy changes and corresponding structures of the first elementary reaction step of OER on different active centers.

# References

[1] G. Kresse;J. Furthmüller, *Phys. Rev. B.* **1996**, *54*, 11169-11186.

[2] J. P. Perdew; K. Burke;M. Ernzerhof, *Phys. Rev. Lett.* **1996**, *77*, 3865-3868.

[3] P. E. Blöchl, *Phys. Rev. B.* **1994**, *50*, 17953-17979.

[4] H. J. Monkhorst;J. D. Pack, *Phys. Rev. B.* **1976**, *13*, 5188-5192.

[5] S. Grimme; J. Antony; S. Ehrlich;H. Krieg, *J. Chem. Phys.* **2010**, *132*, 154104.

[6] J. K. Nørskov; J. Rossmeisl; A. Logadottir; L. Lindqvist; J. R. Kitchin; T. Bligaard;H. Jónsson, *J. Phys. Chem. B.* **2004**, *108*, 17886-17892.

[7] X. Zhao;Y. Pei, *J. Phys. Chem. C* **2021**, *125*, 12541-12550.
